# The Black Box of Nordic Education Held Against the Light of Large-Scale International Assessment Resources—A Critical Commentary

**DOI:** 10.1007/978-3-030-61648-9_15

**Published:** 2020-10-08

**Authors:** Fritjof Sahlström

**Affiliations:** 1 Department of Teacher Education and School Research, University of Oslo, Oslo, Norway; 2 Department of Teacher Education and School Research, University of Oslo, Oslo, Norway; 3 Department of Teacher Education and School Research, University of Oslo, Oslo, Norway; 4 Department of Teacher Education and School Research, University of Oslo, Oslo, Norway; Åbo Akademi University, Turku, Finland

**Keywords:** Black box, Nordic education, Large-Scale International Assessment, Classroom interaction

## Abstract

This book answers the following general question: when it comes to the impact of socio-economic status (SES) on student results in the context of the so-called Nordic model, what can we learn from large-scale international student assessments? The findings presented are not only new and valuable, but they also raise critical questions, some of which I will discuss below.

This book answers the following general question: when it comes to the impact of socio-economic status (SES) on student results in the context of the so-called Nordic model, what can we learn from large-scale international student assessments? The findings presented are not only new and valuable, but they also raise critical questions, some of which I will discuss below.

Being both insightful and problematic is a feature that the chapters in this volume share with much other education research. Whichever way an educational problem, such as equity, is approached, it seems that one runs the risk of getting stumped by the overwhelming complexity of educational processes. However, being insightful and problematic at the same time is what serious research in the field of teaching and learning looks like. As David Berliner (2002) puts it succinctly in *Educational Researcher*, ‘Education is the hardest science of all’ (p. 18). Berliner’s text was written in response to government expectations of education science to deliver so-called hard results. In the article, Berliner (2002) argues vehemently for a more reflexive view of educational science.

Almost 20 years later, I think Berliner still has something to say in relation to the chapters in this volume. ‘The remarkable findings, concepts, principles, technology, and theories we have come up with in educational research are a triumph of doing our damndest with our minds. We have conquered enormous complexity’, Berliner (2002) writes (p. 20). I think he is correct in this observation and that it also pertains to this book and to the many readings I hope it will get.

It is in this context that the volume should be read and appreciated—as a serious, empirical effort to deepen our knowledge of how schooling in Nordic countries contributes to equity and equality. It has its challenges, of which I think the limited attention paid to Nordic contextualisation is the most substantial, but it is still very much a worthwhile read, particularly for anyone interested in how education for all turns out to be more for others and not always the ones we are aiming for.

The volume fits into the category of black box literature; the basic idea here is that some kind of input goes into a system and gets chewed within the inner workings of this system, and then some kind of output comes out at the other end. The system is the black box. The research interest is in the relation between the input and the output, rather than in what goes on under the hood of the system.

The term ‘black box’ might initially bring to mind the crash investigation method, in which technical log data from the black box of an airplane or some other mode of transport is investigated in order to understand why an accident occurred. Reading the volume in this way would be to intentionally misunderstand it for several reasons. One of them is that black box investigations of this kind are carried out when things fall to the ground or sink. In general, Nordic education has not crashed. In fact, it is doing quite well, as it continues to fly the post-modern skies of a rapidly changing world whilst encountering some turbulence on the way.

This brings us to the related but much more difficult approach to understanding processes, namely, trying to determine not how and why things crash but how they actually work. In the case of this volume, how does education for all in Nordic countries stay in the air, where is it heading, and why? This is the pursuit of the chapters of this volume, and their shared chosen approach is to dig deeper into the findings of international large-scale assessment studies.

Understanding how things work is much more difficult than understanding why they fail. Despite doing their best with what is available and succeeding well in doing so (more about this later), large-scale assessments cannot really claim to access any inner workings of education. Rather, what we have at hand are studies which, in essence, are refined and sophisticated input–output models put to work. The chapters are black box studies in the sense of systems engineering and economic production functions, in which inputs (e.g. money spent per pupil, facilities, teacher qualifications) go into a box called *schools*, and outputs emerge (e.g. test scores, skilled and knowledgeable high school graduates, humane and community engaged adults). Still, the matter of how inputs are converted into outputs within the black box continues to be unknown (Cuban, 2016).

The research is focused on illuminating the effectiveness of Nordic education in greater detail than what has been done in prior research in relation to matters of equity and equality. This is a fine ambition. In Chap. 10.1007/978-3-030-61648-9_2, the authors present this ambition quite explicitly in response to criticism of large-scale assessment:

Only the use of standardised and internationally comparable instruments makes it possible as objectively as possible to assess the performance of school systems and thereby give a non-biased indication of the level of achievement of equity and equality in the educational system, at least in part. The SES of students as a psychometric construct is defined in various large-scale studies using a conglomerate of different variables and is linked to students’ performance in order to obtain scientifically justified statements. (Buchholtz, Stuart & Frønes, 2020, p. 35)

In addition to being ambitious, it is also quite bold to state at the outset that the chosen instruments in the chapters are the **only** way forward. In principle, there are some quite considerable doubts in relation to any approach being the **only** one within educational sciences. Many researchers, also within the quantitative paradigm, would disagree in principle. So do I, but there is no point in extending that argument here; the arguments are well known. No educational research approach can claim an epistemic monopoly, and even without monopoly claims, no educational research approach can claim to be objective. However, overseeing these principal problems is worthwhile to get to other and more interesting empirical problems. Of these, there are many. Here, I will focus on two aspects: (1) the understanding of the Nordic model displayed in the chapters and issues pertaining to this matter and (2) the understanding of the inner workings of the systems that are claimed to produce the results compared in the anthology.

## The Nordic Model in the Chapters

In my reading, the book is interested in the following two questions: Does participation in education reduce or widen socio-economic differences? Is there a difference between Nordic countries in the amount of this widening or weakening of difference?

The basic conceptual model is that SES-related differences are the dependent variables, and the variations between countries are the independent variables. For this kind of comparison to be meaningful, the underlying premise is that the Nordic model is stable enough and shared enough to be considered similar in all Nordic countries. Furthermore, within this model, it is presumed that the aim in relation to equity and equality is the same, which would be to reduce SES-related differences. The question to consider, therefore, is whether there is country variation.

This is simply put, but to be as clear as possible.

Reflecting their shared methodological paradigm, the authors, in general, are interested in and skilled at data analysis, and the chapters reflect their depth of knowledge of quantitative analysis. Understandably, issues of contextual and conceptual framing are not developed at the same level of sophistication. The chapters all have an initial section on equality and equity, but fairly little is said in these sections that would substantiate the so-called Nordic model. The not-so-extensive analysis of the Nordic model is also evident in the limited amount of contextualised reasoning of the differences found. What are the consequences of the Nordic model, what are the country consequences, and what is something else? How can one discern the first two from the third? For a volume focusing on the Nordic model, there is surprisingly little discussion of the actual model.

The limited attention given to the Nordic model as such could have been balanced by an empirical analysis at the Nordic level, in which breadth in Nordic empirical work would be a counterweight to limits in model-level contextualisation. Despite some chapters succeeding in doing so, this is not fully the case. As shown in Table 15.1, three of the eleven empirical chapters (Chaps. 10.1007/978-3-030-61648-9_4, 10.1007/978-3-030-61648-9_8 and 10.1007/978-3-030-61648-9_14) contain all Nordic countries, i.e. Denmark, Finland, Iceland, Norway and Sweden. These three chapters are the only ones that include Iceland. Chapter 10.1007/978-3-030-61648-9_5 includes the remaining four countries. One chapter, Chap. 10.1007/978-3-030-61648-9_12, includes the three remaining countries, three chapters (Chaps. 10.1007/978-3-030-61648-9_6, 10.1007/978-3-030-61648-9_7 and 10.1007/978-3-030-61648-9_11) are two-country comparisons and three chapters (Chaps. 10.1007/978-3-030-61648-9_9, 10.1007/978-3-030-61648-9_10, 10.1007/978-3-030-61648-9_13) include Norway only. Norway is the only country included in all chapters. The tally for the countries is as follows: Iceland: 3 chapters, Finland: 4 chapters, Denmark: 6 chapters, Sweden: 7 chapters and Norway: 11 chapters.

**Table 15.1:** Chapters and included Nordic countries

Chapter	Author/s	Countries
10.1007/978-3-030-61648-9_4	Julius K. Björnsson	Denmark, Finland, Iceland, Norway, Sweden
10.1007/978-3-030-61648-9_5	Kajsa Yang Hansen, Jelena Radišić, Xin Liu and Leah Natasha Glassow	Denmark, Finland, Norway, Sweden
10.1007/978-3-030-61648-9_6	Anubha Rohatgi, Jeppe Bundsgaard and Ove E. Hatlevik	Denmark, Norway
10.1007/978-3-030-61648-9_7	Trude Nilsen, Ronny Scherer, Jan-Eric Gustafsson, Nani Teig and Hege Kaarstein	Sweden, Norway
10.1007/978-3-030-61648-9_8	Ronny Scherer	Denmark, Finland, Iceland, Norway, Sweden
10.1007/978-3-030-61648-9_9	Guri Anne Nortvedt, Karianne Berg Bratting, Oksana Kovpanets, Andreas Pettersen and Anubha Rohatgi	Norway
10.1007/978-3-030-61648-9_10	Ole Kristian Bergem, Trude Nilsen, Oleksandra Mittal and Henrik Galligani Ræder	Norway
10.1007/978-3-030-61648-9_11	Jelena Radišić and Andreas Pettersen	Sweden, Norway
10.1007/978-3-030-61648-9_12	Tove Stjern Frønes, Maria Rasmusson and Jesper Bremholm	Sweden, Norway, Denmark
10.1007/978-3-030-61648-9_13	Ragnhild Engdal Jensen	Norway
10.1007/978-3-030-61648-9_14	Hildegunn Støle, Åse Kari H. Wagner and Knut Schwippert	Denmark, Finland, Iceland, Norway, Sweden

Against the background of the focus on Nordic equity, the distribution of countries is a little surprising, particularly when considering the highly empirical character of the research presented.

The skewered inclusion of empirical cases would not necessarily have to be problematic. One could argue, in principle, that the Nordic model is robust enough to be present in all countries and that because of the availability of Norwegian data to Norwegian researchers, it makes sense to use Norway more than what is arithmetically plausible. Furthermore, one could argue that the variation in comparative combinations is warranted against the background of the subject matter to be analysed, and that the arguments for this could have been presented in each of the chapters and in the introduction. As mentioned, the authors of the volume have decided not to fully pursue this possibility of extending the discussion of the Nordic model. It is also the case that the empirical comparative possibilities have not been fully explored. There are no chapters in which the differences and similarities found are discussed at a more analytical level than the mention of and possible short discussion of intra-country differences.

In combination, the scarcity of both theoretical and empirical discussion of the Nordic model sometimes hampers the scope of the claims made on the basis of the studies carried out, leading to chapter conclusions that are not always as focused on either the Nordic model or country differences as would have been possible. As a consequence, the book is not as succinct as it could have been in relation to the Nordic model and whether its continued existence is an empirical or conditional question.

In the interesting first chapter of the book, written by Nils Buchholtz, Amelie Stuart and Tove Stjern Frønes, there is a thorough discussion of the concepts of equity, equality and diversity from conceptual and philosophical points of view. This analysis is reflexive and widely read, and it contributes to the field with valuable insights and points of view. The conceptual discussion is followed by a discussion per country of what is presented as ‘educational policy measures and historical developments in the individual Nordic countries in connection with equity, equality and diversity when dealing with marginalised groups’ (Chap. 10.1007/978-3-030-61648-9_2, p. 25).

Unfortunately, this ambition falls somewhat short, partly as a consequence of an informed choice, in which lack of space is argued to warrant a focus on diversity primarily. Partly, it is also a consequence of what could be argued to be an under-theorising and under-contextualising of the similarities and differences within the Nordic model in relation to reducing socio-economic differences.

There is substantially more to Nordic SES variation than cultural diversity, and both the introductory chapter and the volume as a whole would have benefitted from a more careful discussion of these matters. As an example, in an extensive review article, Dovemark et al. (2018) from the Nordforsk Excellence Center Justice through Education (JustEd). write in *Education Inquiry* about the changes in Nordic comprehensive education, in a special issue dedicated to Nordic education and social justice, In this article, Dovermark and her colleagues argue that deregulation, marketisation and privatisation have implied many changes in Nordic education, in which the idea of a knowledge economy is replacing the previous welfare narrative but in which degrees of change vary in relation to political and historical contexts. Dovemark and colleagues found that the emerging differences within the Nordic model were found mainly in relation to marketisation and privatisation. Norway and Finland continue to have public education markets, whereas Sweden and Denmark provide a wide range of options. The questions of privatisation were dealt with differently in all five countries because in Sweden and the private educational providers for comprehensive schooling have a far more central role in the educational system than in the other countries. The authors argue that the role of profit-making, which has been enabled in Sweden, may be considered one of the biggest changes in relation to the original model of a uniform comprehensive school, contributing to emerging patterns of social differentiation.

I would have appreciated more of this kind of reasoning in this volume. I have a full understanding of the difficulties in trying to achieve this, but to me, it is precisely this dimension that I find interesting. As the volume stands now, the care paid to the quantitative analyses is not fully rewarded with a similarly insightful contextualisation, neither in relation to the Nordic dimension nor in relation to socio-economic differences. Yes, there is variation between the chapters, and there are chapters in which the differences between the countries found in the results section are also followed up by a discussion of both the Nordic dimension and socio-economic aspects (e.g. Chaps. 10.1007/978-3-030-61648-9_4 and 10.1007/978-3-030-61648-9_14). Overall, however, there is an imbalance. This imbalance takes away some of the value of the volume and puts the burden of making conclusions quite heavily on the shoulders of readers.

## How Understanding the Inside Might Add to Input–Output Studies

As already mentioned above, another possible angle of approach to this volume for a critical reader is to reflect on whether the black box approach taken, i.e. the input–output model discussed above, can be a reasonable way of understanding how a system works; knowing the inner workings of the system also matters for how equity and equality are achieved. A 2018 article by Kirsti Klette and her Nordic colleagues sets out to analyse issues of education and justice from the inside, with a review of empirical analyses drawing on video recordings of Nordic secondary classrooms. Being one of the authors, I will repeat the gist of the argumentation in that article here.

Nordic classrooms share a societal expectation that equal opportunities will be provided for all within the framework of comprehensive schooling. Opportunities to engage in meaningful discursive practices and learning activities are considered key factors in high-quality schooling and education around the world. This interactive and discursive view of learning underscores the power of recurring face-to-face interaction and communication amongst peers, in which language, conceptual familiarity and understanding are seen as critical tools for learning (Sfard, 2008).

As for opportunities for student participation in Nordic classrooms, previous research presents a mixed picture. Several studies show that Nordic classrooms provide ample opportunities for students to speak out and influence classroom discourse, more so than in other countries. However, whilst student engagement and student-active ways of working might be key features of Nordic classrooms, there are also differences within and across Nordic countries. Simola, Kauko, Varjo, Kalalahti and Sahlström (2017) describe Finnish classrooms as places where a substantial amount of time is used on individual tasks, with few opportunities for students to talk. Klette and Ødegaard (2015) argue that Norwegian classrooms support student questioning and engagement; however, student utterances are often used for practical and procedural purposes rather than for cognitively demanding enquiries. Analysing Swedish mathematics classrooms, Emanuelsson and Sahlström (2008) use the term ‘the price of participation’ (p. 205) to discuss the relation between the cognitive and communicative aspects of classroom learning that include a high degree of student involvement.

Classrooms today have been connected through extensive digitalisation via laptops, tablets and smartphones, which also bring new and multifaceted possibilities for gaining access to different kinds of content. The rapid and massive connection of classrooms has, to a large extent, been achieved through students bringing their own devices into classrooms. Mobile gadgets, particularly mobile phones, have become a crucial part of the everyday life of young people. Using social media applications, such as Snapchat, WhatsApp, Instagram and Facebook, is a common activity for most of today’s students, both in the classroom and outside (Paakari, Rautio, & Valasmo, 2019).

In terms of student engagement, the phone screen enables interactive participation (e.g. written and visual messaging, liking, sharing, browsing) parallel to the teaching, without directly interfering with the teacher’s presentation and without violating or threatening the overall participation expectations for students in whole-class teaching segments. Students can and do interact with people outside their classrooms. Compared with talking during the lesson, which previously represented the opportunity for peer-to-peer interaction in whole-class teaching segments, the phone presents a significantly smaller disturbance to the teaching. However, phone use also means that students are less accessible for peer talk, and classrooms become less inclusive as interactional spaces (Sahlström, Tanner, & Olin-Scheller, 2019).

The continued use of whole-class teaching at a time of rapid digitalisation makes sense in relation to the participation constraints of whole-class interaction. The individual access for all students to their devices provides them with the opportunity to participate in interactions in parallel with whole-class teaching, in which their opportunities for participation have always been and continue to be limited, without directly disturbing the teaching. For this reason, the *de facto* digitalisation of secondary-school classrooms via students’ mobile phones and laptops seems to conserve rather than change whole-class teaching as a general pattern. Somewhat ironically, the digitalisation of classrooms, which was expected to drive pedagogical change and lead to increased inclusion, seems to have had the opposite effect. There is a considerable increase in student communicative acts within the context of classrooms, but the interactions do not seem to be the kind that straightforwardly supports either learning or equality (Sahlström, Tanner, & Valasmo, 2019).

In general, whole-class teaching as it is currently being practiced in Nordic classrooms does not seem to be particularly conducive to creating equal opportunities for participation. The interactional logic of basic participation frameworks and the turn allocation practices in classroom interactions seem to promote difference rather than equality; this makes it difficult for whole-class teaching to provide what it is aiming for, namely, equal opportunities for all students to develop their communicative and discursive skills and capacities within and beyond their school subjects. Interestingly, there seems to be a dissonance between Swedish and Norwegian teachers’ active encouragement of student engagement and participation, on the one hand, and the persistence of rather stable and teacher-dominated interaction patterns, on the other hand.

In Nordic classrooms, individualised teaching and the presence of digital technology-based 1:1 solutions seem to weaken rather than strengthen social justice and equity. The increased access students have to content unrelated to the classroom has reduced classroom equality, as students have chosen to remove themselves from the learning context. As other studies suggest, individual choice by students in whole-class teaching tends to increase rather than decrease difference (Dalland & Klette, 2016; Österlind, 1998; Sahlström, 1999). Furthermore, new technological devices paired with traditional teaching may actually limit access both to learning-relevant content and to learning-relevant discourse. As students navigate their personal pathways through the Internet in the classroom, the institutional boundaries between the classroom, school and everything else have become blurred.

Within their limits, findings such as these pose some serious challenges for the Nordic welfare society vision of classrooms as core societal hubs for justice and equality. The inner workings of classrooms seem to contain inherent constraints that do not support equitable student engagement. Åse Hansson (2011) makes the argument that teaching seems, in certain ways, to facilitate pedagogical segregation rather than pedagogical inclusion. Furthermore, the way Nordic classrooms have responded so far to the massive digitalisation of society seems to pose further questions rather than provide the needed answers—questions such as what do we need classrooms for after all, and what should teachers and students be doing in them?

## The Most Difficult Science of All

The reason for spending as much space as above on the organisation of classroom interaction is to point out that if one is interested in what happens in the black box of Nordic education as this volume indeed is, then it seems to make sense to recognise that the teaching that is supposed to mediate equity and equality seems to be rigged towards mediating the opposite. The chapters in the volume do not and should not be expected to try to account for the inner workings of the black box in the sense of paying attention to teaching and learning processes as such because the chosen focus is elsewhere. This is understandable, as the focus is on what can be learned from large-scale international assessment studies. As with any choice, however, something is also lost when a certain method and focus have been chosen.

To be fair, from a scientific point of view, the choices made by researchers approaching the classroom from within too often implies the exclusion of the possibility of considering the relevance of analysis from other perspectives, such as international large-scale assessment studies. At a general level, results at the level of PISA rankings and other easily available findings are quite often used for contextualisation, but engaging in analysis and dialogue beyond one’s own paradigm is uncommon. As one of the many possible examples, in the field of policy analysis, certain empirical matters are sometimes addressed with a quite light hand, resulting in excellent historical and structural contextualisation but with rather sweeping treatments of empirical findings. At the other end of the spectrum, within interaction-focused classroom research, it is quite common to have lengthy discussions of interactional structures inside schools but with very limited or no discussion of how certain structural features come into play at the individual level.

To a certain extent, these challenges are caused by education indeed being the most difficult science of all, as mentioned in the introduction (Berliner, 2002). Despite the bold statement made by the authors of this book, seeing international large-scale assessment as the only and objective instruments (page 35) of approaching equity in education research, I think that the inclusion of a commentary chapter from such a different paradigm as my own tells a different story. To me, the volume benefits from being read in line with Berliner (2002), who writes that ‘… ethnographic research is crucial, as are case studies, survey research, time series, design experiments, action research, and other means to collect reliable evidence for engaging in unfettered argument about education issues. A single method is not what the government should be promoting for educational researchers. It would do better by promoting argument, discourse, and discussion’ (page 20).

I think all the chapters in this book contribute to precisely the kind of argument and discussion Berliner asks for. More work is needed quickly. Research and learning are slow processes, whereas reality is fast. One of the aspects not discussed much in the volume is digitalisation and how digitalisation is related to the Nordic model, education and SES. In the last 10 years, human sociality has been transformed by the opportunities provided by screen interaction. The COVID-19 pandemic has further underscored that the four walls of the equality-constructing classroom we used to take for granted need to be seen in a different light. The walls have not crumbled but have become permeable, allowing for a literal flow of content in and out of what quite recently used to be closed spaces. If and how these new dimensions of education matter are for us in educational research to find out. When doing so, we will be in good company with, amongst others, the authors of this volume. This, I believe, is something to look forward to and has been made much easier by the work done in this volume.

## References

[CR1] Berliner D (2002). Comment: Education: The hardest science of all. Educational Researcher.

[CR02] Buchholtz, N., Stuart, A., & Frønes, T. S. (2020). Equity, equality and diversity—Putting educational justice in the Nordic model to a test. In T. S. Frønes, A. Pettersen, J. Radišić, & N.Buchholtz (Eds.), *Equity, equality and diversity in the Nordic model of education *(pp.13–41). Cham, Switzerland: Springer.

[CR2] Cuban, L. (2016, September 2). *Inside the black box of the classroom*. https://larrycuban.wordpress.com/2011/10/16/inside-the-black-box-of-the-classroom/

[CR3] Dalland CP, Klette K (2016). Individual teaching methods: Work plans as a tool for promoting self-regulated learning in lower secondary classrooms?. Education Inquiry.

[CR4] Dovemark M, Kosunen S, Kauko J, Magnúsdóttir B, Hansen P, Rasmussen P (2018). Deregulation, privatisation and marketisation of Nordic comprehensive education: Social changes reflected in schooling. Education Inquiry.

[CR5] Emanuelsson J, Sahlström F (2008). The price of participation. Teacher control versus student participation in classroom interaction. Scandinavian Journal of Educational Research.

[CR6] Hansson, Å. (2011). *Ansvar för matematiklärande. Effekter av undervisningsansvar i det flerspråkiga klassrummet*. [Responsibility for mathematics learning. Effects of teaching responsibility in the multilingual classroom]. University of Gothenburg, Faculty of Education.

[CR7] Klette K, Ødegaard M, Klette K, Bergem OK, Roe A (2015). Instructional activities and discourse features in science classrooms: Teachers talking and students listening or…?. Teaching and learning in lower secondary schools in the era of PISA and TIMSS.

[CR8] Klette K, Sahlström F, Blikstad-Balas M, Luoto J, Tanner M, Tengberg M, Roe A, Slotte A (2018). Justice through participation: Student engagement in Nordic classrooms. Education Inquiry.

[CR9] Österlind, E. (1998). *Disciplinering via frihet: elevers planering av sitt eget arbete* [Discipline through freedom: Students’ planning of their own work]. Unpublished doctoral dissertation. Acta Universitatis Upsaliensis.

[CR10] Paakari A, Rautio P, Valasmo V (2019). Digital labour in school: Smartphones and their consequences in classrooms. Learning, Culture and Social Interaction.

[CR11] Sahlström, F. (1999). *Up the hill backwards. On interactional constraints and affordances for equity-constitution in the classrooms of the Swedish comprehensive school* (Publication No. 85). Doctoral thesis, Uppsala University. Acta Universitatis Upsaliensis.

[CR12] Sahlström F, Tanner M, Olin-Scheller C (2019). Smartphones in classrooms: Reading, writing and talking in rapidly changing educational spaces. Learning, Culture and Social Interaction.

[CR13] Sahlström F, Tanner M, Valasmo V (2019). Connected youth, connected classrooms. Smartphone use and student and teacher participation during plenary teaching. Learning, Culture and Social Interaction.

[CR14] Sfard A (2008). Thinking as communicating: Human development, the growth of discourses, and mathematizing.

[CR15] Simola H, Kauko J, Varjo J, Kalalahti M, Sahlström F (2017). Dynamics in education politics understanding and explaining the Finnish case.

